# Impact of climate change and variability on traditional farming systems: Farmers’ perceptions from south-west, semi-arid Zimbabwe

**DOI:** 10.4102/jamba.v12i1.742

**Published:** 2020-09-21

**Authors:** Everson Ndlovu, Barend Prinsloo, Tanya le Roux

**Affiliations:** 1 Institute of Development Studies, National University of Science and Technology, Bulawayo, Zimbabwe; 2 Unit for Environmental Sciences and Management, African Centre for Disaster Studies, North-West University, Potchefstroom, South Africa; 3 Department of Security Studies and Management, North-West University, Potchefstroom, South Africa; 4 Communication and Journalism Department, Bournemouth University, Bournemouth, United Kingdom

**Keywords:** climate change, traditional farming systems, perceptions, resilience, climate adaptation

## Abstract

Despite annual climate variability threats, traditional farming in semi-arid Zimbabwe remains entrenched in unproductive, rain-fed agricultural practices. Adaptation strategies by farmers are seemingly failing to mitigate climate impacts, as evidenced by annual crop and livestock losses. Matabeleland South Province was a thriving livestock and small grain-producing province in the 1970s. Today, the province relies heavily on humanitarian assistance from government and humanitarian agencies. Through literature review, observations and focus group discussions with 129 farmers, the qualitative study established the perceptions of farmers around climate variability impacts in the past 20 years in Mangwe, Matobo and Gwanda districts in Zimbabwe. The study (1) analysed changes in climate and weather patterns in the past 20 years; (2) analysed climate impacts on traditional farming systems in the past 20 years in Gwanda, Mangwe and Matobo districts in Zimbabwe; and (3) established farmers’ perceptions, experiences and their climate adaptive strategies. The findings showed that the farmers experienced annual heat waves, protracted droughts, chaotic rain seasons, frost and floods, which led to environmental degradation. Traditional farming systems or practices have been abandoned in favour of buying and selling and gold panning, among other alternative livelihood options, because of climate-related threats and misconceptions around the subject of climate change. Farmers fail to access timely and comprehensive weather forecasts, resulting in annual crop and livestock losses, as decision-making is compromised. Given that the smallholder farming system sustains the bulk of the population in Matabeleland South Province in Zimbabwe, climate education and capital investment is needed to change traditional farmer perceptions about climate change impacts on the farming practices. Increased climate awareness initiatives, establishment of village-based weather stations and the marrying of traditional farming climate knowledge to modern practices are highly recommended to enhance resilience to climate.

## Introduction

Shifting climatic conditions in Africa have dramatic impacts on the livelihoods and food security of farmers who remain reliant on rain-fed agriculture (FAO 2006, 2007). Poverty, food insecurity, water scarcity and environmental degradation characterise arid Southern Africa, including Zimbabwe (Wani, Rockstrom & Oweis 2009). In Zimbabwe, 37% of the country receives adequate rainfall for rain-fed agriculture, yet as much as 90% of the population is dependent on this practice (FAO 2006; Unganai & Murwira 2010). For centuries, rain-fed agriculture has not only sustained food security for many Zimbabweans, but it has also contributed immensely to the country’s industrialisation. However, prolonged droughts and other shocks resulting from climate change and variability have impacted heavily on traditional farming practices such as extensive cattle ranching, semi-pastoralism, cultivating of rain-fed crops like maize, millet and sorghum, crop rotation and intercropping, minimum or no usage of commercial inputs like fertilisers and hybrid seed, and farmers cultivating their own seed, especially in the semi-arid districts of Gwanda, Matobo and Mangwe. The three districts studied have been experiencing the problem of food insecurity almost on a yearly basis and have lost a considerable number of livestock over the years because of climate-related disasters, such as droughts, pests and diseases. Prolonged food insecurity in the districts has attracted the perennial presence of humanitarian agencies running food aid programmes in the past 15 years. It is the continuous presence of humanitarian agencies, which has tended to indirectly promote a dependency syndrome amongst farmers and their dependents in these districts that motivated this study.

Agricultural production in the past two decades (1990–2010) in Matabeleland South Province has been constrained by water scarcity resulting from climate change (Musarurwa & Lunga 2012). Changing rainfall patterns, frequent droughts and flash floods continue to adversely affect both livestock and crop yields. Projected climate impacts on agriculture in Southern Africa are that the productivity of dry land farming, will be hardest hit (Kurukulasuriya et al. 2006). Dry-land farming yields are projected to decline by 20% – 50% by 2050 (IPCC 2007b). Given that more than 90% of dry-land smallholder farmers in the study districts are dependent on rain-fed agriculture, projections are that climate change impacts could be devastating for the farmers if effective climate-smart adaptation interventions are not embraced (Unganai & Murwira 2010).

Smallholder farmer production in semi-arid Zimbabwe constitutes 90% of all agricultural activities (Unganai & Murwira 2010). Farmers involved in rain-fed agricultural activities are in the majority of ‘traditional agriculturalists’ who still practise semi-pastoralism and extensive livestock production, cultivate maize, millet, sorghum and groundnuts during the rainy season and plant only for family consumption. The production practices in this sector (mixed farming) are aimed at sustaining families, with little surplus for sale. Although productivity varies from place to place, dry-land farming remains the major source of livelihood for many of the vulnerable farmers in semi-arid Zimbabwe (Kahinda et al. 2007). The potential for traditional agriculture has been constrained by climate variability, particularly in Matabeleland South Province (IPCC 2014; Tshuma & Mathuthu 2014). As such, future smallholder agricultural productivity in the province in particular, and Zimbabwe in general, is likely to suffer (UN-OCHA 2015).

This article analyses changes in the climate and weather patterns over the past 20 years; discusses climate impacts on traditional farming practices over the past 20 years in Gwanda, Mangwe and Matobo districts in Zimbabwe; and establishes farmers’ perceptions, experiences and their climate-adaptive strategies. The effects and impacts on traditional farming practices are explained from the point of view of farmers.

### Statement of the problem

Annual precipitation levels in Zimbabwe have gone down in the past two decades, with Matabeleland South Province experiencing an annual average of between 350 mm and 500 mm, against a national average of 500 mm – 750 mm (Unganai 1996). The low-lying areas like Matabeleland South, are mostly semi-arid and prone to intermittent droughts. The low-lying areas, including Mangwe, Gwanda and Matobo districts, experience aridity, with unreliable rainfall of between 250 mm and 500 mm per annum (USAID 2015). This amount of rainfall is too low to sustain traditional rain-fed farming practices such as mixed farming. Climate variability and change pose protracted challenges to the productivity of traditional farming practices in the semi-arid districts of Mangwe, Matobo and Gwanda in Matabeleland South Province in Zimbabwe. Subsistence or smallholder farmers, dependent on rain-fed agriculture, have misconceptions about climate change and its impacts on their extensive livestock and crop production practices, as the majority believe that the protracted drought, heat waves, frost and floods are an ‘act of God’. Thus, farmers in the three districts are distancing themselves from doing something about their situation. In the process, the vulnerable farmers continue to lose their livestock, and their crop harvests dwindle on a yearly basis. The impacts have been devastating to both extensive livestock production and cropping practices in the districts, resulting in the depletion of household assets, increased food insecurity, increased levels of poverty and worsening vulnerabilities in rural areas. The continued presence of humanitarian agencies in Mangwe, Matobo and Gwanda districts points to a dependency syndrome on the part of the farmers and their families. Many a farmer has abandoned cropping, knowing fully well that donor-funded humanitarian organisations will intervene through food aid or that the government will distribute agricultural inputs. This is further evidence of low capacities and poor climate early warning systems. Compounding the challenges in the districts are information gaps in terms of climate threats and poor adaptive and mitigation strategies. Poor climate early warning systems mean that farmers fail to make informed decisions for both their livestock and cropping practices, resulting in poor or low climate-adaptive capacities. Farmers still maintain high livestock densities in spite of the dwindling pastures. On the cropping side, famers still plant the water-loving maize seed instead of drought-tolerant seed varieties (Christian Care, 2009; Care International, 2009).

## Review of related literature

Climate change and variability is conceptualised in the literature. The nature and impact of climate threats on the environment and traditional farming systems in semi-arid regions in sub-Saharan Africa are analysed and put into perspective to understand the perceptions of farmers and how they then respond to the various climatic threats. The theoretical framework that underpins the study is described and its applicability to the study highlighted.

### Conceptualisation of climate change and variability and impacts on traditional farming systems

Climate regimes across Africa range from arid and seasonally arid tropical climates to humid equatorial, all with differing degrees of variability and change (Desanker 2001; Ndhlovu 2009; Sokona & Denton 2001). Poverty and poor economic development characterise Africa, hence the continent’s vulnerability to climate hazards. There has been a warming of approximately 0.7 °C over most of Africa in the 20th century, a decrease in precipitation in the Sahel and Southern Africa and an increase in rainfall in east and central Africa (IPCC 2007a; Tadross, Pinto & Lennard 2005).

In Zimbabwe, the concept of climate change is understood as referring to statistically significant variation in either the mean state of the climate or its variability persisting over prolonged periods (ZMD 2007). Changes in the long-term weather patterns caused by natural or external forces – attributed directly or indirectly – define what is meant by climate change (IPCC 2001a). Zimbabwe has documented scientific evidence indicating experiences in extreme and adverse effects of climate change (Ndhlovu & Mpofu 2016). Adverse weather conditions include severe dry spells, cyclones, heat waves and flooding events. These are linked to the impact of climate change and variability. Day temperatures increased by 0.8 °C between 1933 and 1993, whereas rainfall decreased by 10% in the same period (Unganai 1996). Extremes in heat waves, flash floods, protracted droughts and increasing mid-season dry spells are an indicator of climate change, and that climate variability occurs within the broader spectrum of climate change (Gumbo 2006). Therefore, ‘climate change’ and ‘climate variability’ are used interchangeably in this article.

Smallholder farmers in arid Africa have witnessed dramatic climate change and variability impacts on their crop, livestock and livelihoods (FAO 2006, 2007). Protracted droughts, heat waves, late start of the rainy season, poor rainfall distribution, frost and floods have negatively impacted on both livestock and cropping in semi-arid regions. As a result, sub-Saharan Africa and Southern Africa, in particular, are witnessing increasing vulnerability to poverty, protracted food insecurity and environmental degradation (Wani et al. 2009). In arid Zimbabwe, 90% of the population is dependent on rain-fed agriculture, with mixed farming (crop and livestock) being the major livelihood activities (FAO 2006; Unganai & Murwira 2010). Although traditional farming production systems such as semi-pastoralism, intercropping and small livestock production have sustained food security for populations in arid Zimbabwe in the past 30 years, current trends indicate low productivity, even for indigenous breeds, declining grazing, low-quality pasture and seasonal water challenges for both livestock and crop farmers (Ayantude et al, n/d). Climate variability such as delayed start of the rainy season, mid-season dry spells, heat waves and frost droughts have impacted heavily on crop and livestock production practices. Compounding this challenge has been low climate-adaptive and mitigation capacities and weak institutional support arrangements at the community and national government levels. Farmers, for example, still prefer maize, a water-loving crop, to small-grain crop varieties that are drought-tolerant. Weather information from government extension offices is not specific to smaller areas and not packaged in a language that most farmers understand, making it difficult for farmers to interpret and make meaningful decisions from it. For instance, the studied districts in Matabeleland South are remote in terms of radio and television coverage, which is poor. Therefore, farmers do not benefit much from the weather bulletins and other related climate information that is channelled through these media. These are some of the challenges bedevilling Gwanda, Matobo and Mangwe districts in Matabeleland South Province in Zimbabwe that motivated this study.

Traditional farming practices in Matabeleland South Province of Zimbabwe are dependent on rain-fed agriculture. According to Morton (2007), subsistence farming only produces enough to sustain families, with very little surplus for sale because farmers have not bothered to or lack resources to invest and expand their operations. Low productivity, exacerbated by diminishing productive assets, poor or weak early warning systems, climate change misconceptions, poor climate mitigation and low adaptive local capacities continue to negatively impact on smallholder farmers, who are not eager to adopt climate-smart agricultural practices (Conway 2008). Depletion of productive assets such as livestock, land, soils, water, implements, labour and knowledge has resulted in a vicious cycle of poverty, food insecurity and dependency on external humanitarian assistance (Ndlovu 2011). Weak disaster risk-reduction institutional arrangements for climate change at the village, ward, district, provincial and national levels have not assisted in facilitating resilience building of livestock and crop-farming management in Zimbabwe (Government of Zimbabwe [GoZ] 2013). Smallholder farmers are, therefore, vulnerable to droughts, heat waves, mid-season dry spells, frost and floods, and there is a need for concerted efforts by stakeholders to come up with viable mitigation and adaptation strategies to promote resilient farming practices amongst subsistence farmers.

Rainfall regimes are erratic, unevenly distributed and in short supply, leading to crop and livestock failure. According to Coetzee et al. (2016b), the increasing vulnerabilities to mid-season dry spells and droughts, for example, of smallholder farming practices, which support over 80% of the population in Southern Africa, are a major concern that needs urgent attention from all stakeholders. It is clear from this premise that community resilience to climate threats within smallholder farming communities has been eroded over time. This article provides for the conceptualisation of climate change through the perceptions of local farmers. It also provides for the analysis of climate and weather information over the past 20 years in Mangwe, Matobo and Gwanda districts and of the impacts of such weather changes on traditional farming practices in Matabeleland South Province of Zimbabwe.

### Theoretical framework

Climate-related threats such as droughts, mid-season dry spells, flash floods and frost occur within a social, economic and political context or environment (Wisner et al. 2004). The study is underpinned by climate change adaptation and mitigation strategies, which are informed by disaster risk reduction, social protection and livelihood approaches to ensure improved disaster preparedness and resilience for vulnerable communities, including vulnerable traditional or smallholder farmers. The conceptual framework informing this study is embedded within the climate change adaptation initiatives aimed at promoting robust and sustainable early warning climate strategies for poor traditional or subsistence farmers, who are dependent on rain-fed agricultural practices for their livelihoods. The resultant product of a robust early climate communication strategy is increased resilience and adaptive capabilities of local populations, including subsistence farmers in Matabeleland South Province of Zimbabwe.

The Sustainable Livelihoods Framework (SLF) by Department for International Development (DFID 1999) was adopted for this study. The framework measures the levels of resilience amongst traditional farming communities, with a view to improving capacities and adaptation capital across the individual household and institutional spheres. The SLF (DFID 1999) provides for resilience building from a three-strata approach: (1) as a development objective, (2) as a set of principles and (3) as an analytical framework (Farrington & John 2001).

From a development objective, the SLF facilitates evaluation of whether poor, subsistence farming communities and those excluded from the mainstream economy have adequate means of livelihood that promote a decent and dignified way of life. Although a traditional farmer’s choice of livelihood strategy influences his or her level of food security and income, it is also possible that the farmer’s level of food security can also influence which livelihood strategy he or she adopts. As a set of principles and an analytic framework, the SLF will be used to determine the human, social, natural, physical and financial capital (assets) that traditional farming systems have or do not have to appreciate their climate-adaptive strategies and capacities or lack thereof (DFID 1999; Wright, Kristjanson & Bhatta 2012). Questions abound as to whether traditional farmers have the human capital (skills and knowledge) to work the land and strategise around climate challenges. Do their social networks remain robust? Do they have the financial capital to invest in climate-smart agricultural practices and to what extent do their land holding and improvements help them to mitigate climate challenges? Once these assets are mapped or determined, it would be easy to suggest alternative interventions to revive or improve traditional farming systems in the era of climate uncertainties.

These livelihood assets determine the farmer’s level and path of development in the wake of climate change and variability (Wright et al. 2012). The framework therefore underpins this study on traditional farming communities in Gwanda, Matobo and Mangwe districts in Matabeleland South Province in Zimbabwe. The community or household’s livelihood assets or capital, activities and access to these, mediated by institutional arrangements and social relations (transforming structures and processes), together determine the traditional farmer’s resilience (bouncing-back capacity) and well-being in the three study sites. A sustainable livelihood maintains and improves people’s capabilities and assets, in the present and future, without damaging the existing natural resource base (improved livelihood outcomes) (DFID 1999). Improved livelihood outcomes include more income, increased well-being, reduced vulnerability, improved food security and more sustainable use of the farmer’s resource base. With improved financial base (income), traditional farmers will be in a position to invest in water point development for irrigation purposes, purchase drought-tolerant seed varieties and livestock, afford insurance for both their crops and livestock (risk mitigation) and also invest in other forms of climate-smart agricultural technologies such as solar-powered water engines and greenhouses. With generally improved assets for farmers, it is envisaged that poor traditional farmers can withstand climate threats such as droughts, long dry spells and frost and get on the path to climate resilience.

### Strategies for climate change and variability available to traditional farmers

Frankenberger et al. (2000) emphasise the importance of absorptive, adaptive and transformative capacities, the very capacities needed to transform the perceptions of traditional farmers in the studied districts. These include access to productive (proxy wealth indicators) assets (physical, human and natural), individual or household livelihood options and institutional arrangements (social and financial), as well as preparedness, prevention, response and recovery activities formulated to achieve improved community outcomes in response to climate-related shocks.

In relation to traditional farmers, physical assets would include land holding (size and tenure), dip tanks, dams, boreholes, holding pens and communication infrastructure, whereas natural assets would include rivers, pasture/veldt, forests, soils and flood plains. ‘Human assets’ refer not only to able-bodied men and women providing labour to till the land and manage livestock, but also to the skills and knowledge needed to facilitate climate adaptation and mitigation. ‘Social assets’ describe community arrangements and structures that facilitate improved governance, networks, safety nets and related structures that promote community bonding and assistance in times of climate stressors and disasters, and the financial aspect describes both household and community wealth creation resources and incomes, including both liquid and fixed assets. The SLF, adapted for this study, informs how access and usage of the forms of assets can improve traditional farming productivity, resulting in improved food security and climate resilience amongst traditional farming communities, hence its appropriateness for this study. The SLF was also used to identify opportunities available in terms of the development of these assets within communities and households. For traditional farmers in the study districts, food security is under threat; hence, models that intervene to change the status quo for the better are well placed to move communities from their vulnerable status to a more robust and capacity-enhanced status. Through awareness and education, by using the SLF, traditional farming communities in the study sites should be able to treat their small individual plots, maximum three hectares, as economic units that can be transformed into income-generation units and insurance against climate hazards. Other opportunities include enhanced climate information dissemination through improved climate early warning systems, farmer-to-farmer learning transfers, climate knowledge banks and their improved utilisation for the benefit of the whole community and adoption of climate-smart agricultural technologies such as conservation farming, improved water harvesting (field trenches or infiltration pits) and increased use of irrigation technologies.

Vulnerabilities largely manifest at the local level, implying that climate-adaptive strategies should be locality specific (FAO 2008; Fewsnet 2009). Adaptive strategies should enhance food security and water availability, combat environmental degradation, reduce decay in biodiversity and improve on ecosystem services. These challenges are a direct result of climate variability (prolonged droughts, intermittent droughts and delayed rainy season) and poor environmental management on the part of the farming communities resulting from lack of climate education and awareness. In Central America and sub-Saharan Africa, raising awareness of climate change impacts amongst both local farmers and policymakers has been tried out with very minimal success given the resource challenges (Thorlakson & Neufeldt 2012). Improved access to information is the hallmark of transformative behaviours. Investment in the development of various assets as expounded in the SLF, that is, if these are found to be lacking amongst traditional farmers in the three study districts, will enhance water-harvesting techniques, climate-smart agriculture technologies, education and training, among other assets that are likely to build climate resilience amongst traditional farming communities.

Traditional farming communities and policymakers need climate information to make meaningful farming decisions to enhance food security. Strategies such as agro-forestry, conservation farming, focus on water harvesting technologies, use of indigenous knowledge for both livestock and crop management and climate forecasting are likely to increase climate adaptation and resilience. For Zimbabwe, and indeed Matabeleland South Province, both government and the non-governmental organisation (NGO) sector, including the United Nations agencies, have promoted rainwater harvesting, construction of water storage and irrigation systems as a way of reducing dependency on rainfall, albeit with very little coverage owing to resource constraints and the economic crisis in the past decades (Government of Zimbabwe 2016). For the few farmers who have adopted conservation farming techniques, for example, success rates in terms of improved harvests have been reported, particularly for small grains such as millet and sorghum in Matobo and Gwanda (CCare 2012). CCare (2012) reports yield increase from 1 to 3 tonnes/hectare for small-grain crops. Whilst reporting low adoption rates for climate-smart agriculture, describes phenomenal success stories on the increasing use of contour ridges, water-harvesting trenches in fields and the use of small grains, particularly for women farmers. Also being promoted is the diversification of crops, with emphasis on small grains such as millet and sorghum, including small livestock such as goats, chickens and rabbits. The private sector has promoted risk transfer through crop and livestock insurance schemes for extreme weather events to help poor households cope with droughts, floods and pests. Econet Wireless, a telecommunication company in Zimbabwe, is working with traditional farmers to insure their livestock and crops (Van Niekerk, Ndlovu & Chipangura 2015). The effectiveness of these climate-smart adaptive and resilience construction initiatives are yet to be evaluated and appreciated.

## Research methodology

Provided in this section is a detailed description of the study site, research approach, data collection and sampling procedures, data analysis and ethical considerations.

### Description of the study site

**FIGURE 1:**
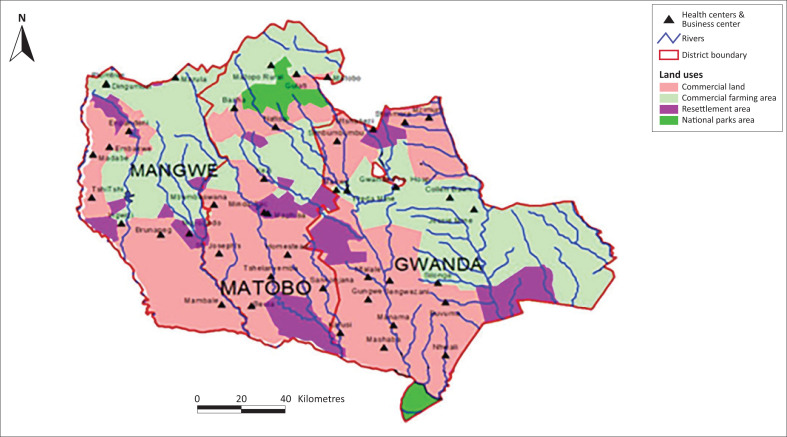
The map of Matabeleland South showing Mangwe, Matobo and Gwanda districts.

Figure 1 shows a map of the three study districts in Matabeleland South Province of Zimbabwe (Mangwe, Matobo and Gwanda). The larger portion of the three districts is under communal farming (traditional farming systems). Over 75% of the most vulnerable farmers eke out a living through traditional farming practices (mixed farming) that are depended on natural rainfall. Farmers in the three districts, who depend on rain-fed agriculture, have witnessed diminishing crop and livestock yields since 2000 as a result of intermittent and unreliable rainfall. The unpredictable weather patterns resulting from climate change and variability have left farmers exposed to climate stressors. Such exposure has compromised traditional farming calendars, resulting in increasing food insecurity. Climate-related stressors such as water scarcity, diminished pastures, pests and disease outbreaks for both crops and livestock and increasing poverty have compounded food insecurity in the study sites (WVI 2015).

The three districts (Figure 1) have uniform climate and weather patterns. They all have suffered protracted droughts, heat waves, frost, floods, intermittent and unreliable rainfall patterns, and mid-season dry spells in the past 20 years. The subsequent related impacts have been water scarcity, crop failure and diminishing pastures, resulting in disease outbreaks and the death of livestock. Loss of productive assets has further exposed traditional farmers and their households to future shocks and poverty. The cycle of poverty has continued in the past 20 years, evidenced by the reduction in household productive assets. Farmers sell their productive assets like ox-drawn ploughs and carts to buy food as part of their adaptive strategies. Increasing outward migration, particularly of the active population, and adoption of alternative livelihood options such as gold panning and prostitution have surfaced in the study sites. Farmers are seemingly failing to change their situation largely because of low or poor climate-adaptive and resilience capacities and strategies, given their low levels of climate awareness and knowledge.

### Research approach

This qualitative study sought to describe and explain farmers’ views and perceptions about climate change and variability within the context of traditional farming systems in Mangwe, Matobo and Gwanda districts in Matabeleland South Province in Zimbabwe. A qualitative study design aims to answer the questions *what, how* and *why* as it better expands on preliminary knowledge about a specific research problem. The local farmers in the three districts have unique knowledge and understanding that should never be overlooked, as they are very likely to contribute to solving some of the climate variability challenges already faced by subsistence farmers. A qualitative research approach facilitates understanding of the perspectives or meanings from the point of view of the study participants. Such designs provide data on participant experiences, their needs and considerations (Stake 2000). The approach was applicable in this study as it sought to understand insights on the experiences and perspectives of farmers in as far as they relate to climate change, variability and the impacts on their livelihoods. The qualitative research approach aspect touched on factual evidence (observed and communicated by participants) and recorded observations, and these facilitated the categorisation of responses into thematic areas for analysis (Creswell et al. 2007). This allowed for conceptualisation, theorising from responses and categorisation of the various climate-change/variability, resilience and vulnerability themes (Allen 2003). This approach explored the subject in more depth and led to a holistic understanding and thorough analysis of the issues on the ground, as observed by Terre Blanche, Durrheim & Painter (2006).

### Data collection procedure

A literature review was used to complement focus group discussions on farmers’ perceptions about the impacts of climate change and variability on their traditional farming practices. Focus group discussions provide for an in-depth understanding of social issues as they are participatory in nature (Morgan 2002). According to Cornwall and Jewkes (1995), this is a bridging strategy for scientific research and local knowledge. Focus group discussions provide for the capturing of wider views. They are cost-effective, and the group is very likely to be representative of the broader community, though members may not express their honest personal views about the subject under discussion. This is highly pronounced when participants’ thoughts are at variance with others. To counter focus group discussion shortcomings, extension officers from government departments and NGOs participated in the discussions. They also became key informants as they answered and elaborated on certain key issues outside the focus group discussion set-up.

To qualify the views generated from the farmers and literature, the study employed observation as another data collection tool. Observations are a direct and accurate method of data collection (Singh 2017). The mere fact that in qualitative studies the researcher is an instrument, and physically present, means that one does not have to rely only on participants to report on events and social issues. Real-life situations are assessed, and surveys are very likely to be conducted in inappropriate and impossible environments (Allen 2017). The observed variables included the general state of water sources, grazing land, livestock and crop fields, the general appearance of the environment and forests, and general community actions. The literature review was based on secondary data sources that included academic books, journal articles, climate policies, Intergovernmental Panel on Climate Change (IPCC) reports, online articles and NGO reports on climate change and variability impacts on livelihoods.

### Sampling procedure

A total of 12 focus group discussions (4 per district) were conducted with 129 participants from the three districts. Subsistence farmers were drawn from the extension officers’ registers in the three studied districts. An equal number of farmers were earmarked from each district, drawn out of a hat after allocating numbers to individual farmers. A total of 129 farmers participated in the study, and these comprised 12 agricultural extension and NGO officers, 37 farmers in Matobo District, 44 farmers in Gwanda District and 36 in Mangwe District. Although 40 farmers per district were targeted, some failed to present themselves on the day of discussions. The extra four participants in Gwanda were senior government officers because Gwanda is the provincial capital for Matabeleland South Province. Participants were conveniently sampled to ensure that those farmers who had been practising farming in the districts for more than 20 years participated in the study. Such participants were considered as having adequate historical memory (knowledge of past climate disasters, experiences) and expertise on climate threats and change in the study districts and were known by the extension officers.

### Thematic analyses

Research themes and patterns from recorded immediate thoughts, reactions and interpretations were identified and captured during data collection. The general themes included knowledge of climate change and variability, climate change impacts on crops and livestock, experiences of climate change, individual and institutional responses or strategies on mitigating climate change and variability, challenges of climate change in the past 20 years and possible solutions proposed by the farmers themselves, government and its developmental partners. The responses gathered through the focus group discussions, observations and literature review were categorised into the various thematic areas identified, and these were imbedded into the research questions. The responses were manually analysed by using the various themes established. The recorded responses were transcribed and later manually analysed.

### Ethical consideration

Approval to conduct the study was obtained from the North West University-Research Ethics Regulatory Committee (NWU-00130-15-A7).

Research ethics, according to Resnik (2015), refer to the right or professional way of doing research. They speak to morals and rules for doing right or wrong. Ethics provide a code of professional conduct in research, lest one does harm to study participants and items. In other words, ethics speak to acceptable behaviour to promote research aims and to minimise errors. Resnik (2015) further argues that ethics provide a framework for accountability on the part of researchers, given the competitive nature of research in modern times. Permission and authority to collect data were sought from the relevant stakeholders and authorities through a clearance letter from the North West University, as the study was part of the university studies. The research team, through engagement, cultivated rapport with the district administrators and chief executive officers of the rural district councils, as well as with the chiefs, who are the entry points and custodians of culture in their respective areas. Informed consent was obtained from the respondents, and an enabling environment was created, where all participants felt free and content to participate. No participant was coerced to divulge information or promised incentives for participation. The researchers facilitated mutual understanding with the respondents. Confidentiality was maintained throughout; no participant names were recorded, except where consent was given. High professional standards were upheld, and the findings were reported in a complete and honest manner without misrepresentation. Language experts to interpret the local languages, Kalanga and Sotho, were among the enumerators and were retired teachers drawn from the local communities. The study was also approved by the university’s ethics committee to ensure that it adhered to ethical guidelines for research.

## Results and discussions

Through the literature review, observations and focus group discussions, the study established the farmers’ perceptions, experiences and climate-adaptive strategies; analysed changes in climate and weather patterns in the past 20 years; and assessed climate impacts on traditional farming systems in the past 20 years in Mangwe, Matobo and Gwanda districts in Zimbabwe. The effects and impacts on traditional farming systems are explained in this section from the point of view of the local farmers. A detailed discussion of the results follows.

### Traditional farmers’ perceptions of climate change in semi-arid Zimbabwe

In Matabeleland South, subsistence farmers had varying perceptions about climate change, as noted during the focus group discussions. Farmer A (aged 55), from Maninji Ward in Mangwe District, narrated climate change as reduced rainfall and high temperatures. He noted:

‘The 2014/15 cropping season was the worst as I used to sleep outside the house at night because of the excessive heat wave due to very high temperatures. Our crops and livestock succumbed to the heat and moisture stress as a result of low rainfall and mid-season dry spells.’ (Farmer A, 55 years, Maninji Ward, 2017)

The implication of these observations by farmers was that climate change caused food insecurity, there was increasing food insecurity as a result of crop failure and the death of livestock. Some farmers abandoned crop cultivation in favour of off-farm livelihood options like buying and selling of meal-meal and other basic food commodities, livestock supplementary feed and vegetables. Gold panning across the three districts had replaced close to 45% of farming activities as it attracted both the young and old. Thus farming activities suffered as labour shifted towards alternative livelihood activities like gold panning. Increasing food insecurity attracted donor-driven food aid programmes to mitigate the acute food shortages in the districts. Increasing humanitarian interventions then promoted a dependency syndrome among traditional farmers and their households, and many waited upon humanitarian agencies to provide food. Some farmers felt that climate change was a challenge to predict in their respective communities. In Matobo District, Farmer B viewed climate change as ‘unpredictable changes in seasons and weather patterns’ (aged 60, Malaba Ward, 2017). According to the farmers, the unpredictable weather patterns interfered with farmers’ known planting calendars, as planting dates shifted. Such disturbances to the farming calendar contributed to reduced crop hectarage, resulting in food shortages because of delayed cropping or non-cropping. Farmers in Matobo and Mangwe districts opined that low precipitation and high temperatures would result in reduced productivity within traditional farming practices, a view supported by Hotir (2011). Over two consecutive cropping seasons (2015/2016 and 2016/2017) across the three districts, the researchers observed annual trends of decreasing precipitation and increasing temperatures. This was confirmed by the farmers, who mentioned that rainfall in the districts was erratic, unpredictable, poorly distributed, of low intensity and short-lived. Responses from the focus group discussions also pointed to delayed rains that would start around January, instead of starting in the month of October in the previous year, and ending around March instead of April. Confusion in the cropping dates befell most farmers as they stuck to their original traditional planting dates, in spite of weather and climate change. The changing weather patterns did very little to motivate innovation amongst subsistence farmers, who remained stuck in traditional practices, possibly as a result of lack of information and climate change awareness. The lack of financial capital for the majority of farmers meant that they could not invest in water source development to address acute water shortages in their cropping systems, because the majority of farmers are poor (DFID 1999). Also stated by some farmers was the shortening of the farming seasons, which made effective planning a challenge to the farmers. Farmer C from Gwanda District noted that ‘dramatic shift in the seasons over the past 20 years, protracted dry spells, periodic heat waves and flooding were now common’ (aged 49, 2017). Across the three districts, the farmers spoke of frost and prolonged winter seasons as signs of climate change. Frost and prolonged winters impacted on late cropping and livestock production systems because livestock succumbed to the low temperatures. However, the farmers distanced themselves from contributing to climate change. Some farmers were of the view that the abandonment and neglect of their customs and belief systems and the adoption of ‘foreign’ religions were contributory factors to climate change. Through Focus Group Discussions (FGDs), the farmers noted that many communities no longer prayed for the rain at the rain-making shrine in Matobo District, as they had converted to Christianity. The elder farmers felt that the conversion to Christianity violated their traditional cultural and religious practices; hence, they believed that God was angry with them, as evidenced by protracted droughts and other extreme weather events, such as floods and pests.

Farmers in Mangwe and Gwanda mentioned that climate change and variability influence land degradation. Borrowing from their indigenous knowledge, farmers indicated that the disappearance of certain types of vegetation species such as *mutopi* and *chithamuzi* was an indicator of climate change. Farmers in Gwanda cited an increasing frequency of destructive tropical cyclones in the district, like Cyclone Eline in 2000 and Cyclone Dineo in 2017. From their perspective, tropical cyclones were now a common phenomenon, compared with some 20 years back. Some farmers blamed the village heads and traditional spiritual elders for failing to appease the ancestral spirits, who were said to be very angry with communities that had taken Christianity on board and shunned their traditional worship systems. The belief of the farmers was that embracing Christianity was the reason for the recurrence of droughts and related climate change manifestations, resulting in crop failure and increasing food insecurity. However, this claim was difficult to prove as it touches on spirituality and lack of climate information. They also blamed their spiritual elders for failing to facilitate rainmaking ceremonies and at the Njelele Shrine, in Matobo district, as was the practice by their forefathers, hence the protracted droughts and destructive cyclones. According to the farmers, their *Mwali* [*God*] in Njelele would provide rains at the most appropriate time, if communities paid their respects and appeased the gods annually, to avert climate threats. However, such perceptions by some farmers did not provide a strong basis for causing changes in climate and weather patterns. Communities in the three districts used the Sacred Mountain (Njelele) in Matobo as their spiritual worship centre and, therefore, shared some traditional beliefs. Matobo farmers added that the drying up of wetlands and the decay of certain animal and tree species, which included certain foliage for animals, was further evidence of climate change. Lack of knowledge of climate change and variability was found to be the reason for farmers hiding behind ‘spiritual’ connotations to explain their predicament – increasing food insecurity and poverty. Such views also border on a lack of collective social responsibility, and they tended to play down the social capital or assets as expounded by DFID’s (1999) SLF.

### Effects of climate change on traditional farming practices

As expressed during the FGDs and also witnessed during observations and drawing from the literature, worsening food insecurity, reduction in annual rainfall amounts, protracted droughts, shifting seasons and destruction of farming infrastructure were some of the effects of climate change on traditional farming practices. The effects of these climate variability proxy indicators on traditional farming systems are fully explained in the subsequent subheadings.

#### Climate change can worsen food insecurity

Dependence on the water-loving crop maize, keeping of large herds of cattle, semi-pastoralism and adherence to ‘normal planting dates’ in spite of changes in weather patterns can no longer meet the food requirements of communities in Matabeleland South Province. Changing weather patterns have negative consequences for sustainable development in agriculture and land use, particularly for the vulnerable dry-land farmers. Inference from focus group discussions pointed to a situation where rain-fed cropping, for example, is becoming less and less sustainable in terms of ensuring improved food security for subsistence farming communities. Droughts have become a recurrent feature in Zimbabwe in the last two decades, particularly in agro-ecological regions IV and V, resulting in farmers missing their dry planting dates and related livestock activities, even as observed by Unganai and Murwira (2010). Farmers in the three districts posited that ploughing and cropping dates or calendars were missed due to uncertainties in the start of the rain season. As a result, some farmers stated that they had abandoned crop farming for non-farming livelihoods such as gold panning because of perennial droughts. The resultant effect has been increased food insecurity and decay of their livelihoods. Early wilting of crops resulting from droughts (mid-season) translated to increasing food shortages in the three districts. Farmers observed over the years that droughts also meant reduced pasture, water scarcity (livelihood natural assets according to the SLF), the emergence of new livestock diseases and subsequently the death of livestock, an alternative livelihood for farmers in the study sites. Farmers opined that given their lack of sustainable incomes, they failed to respond effectively to shocks, trends and seasonal climate disturbances and could not afford to purchase livestock medication. Agricultural extension officers acknowledged that government had no resources to support the farmers in terms of dipping chemicals and supplementary livestock feed, an indication of decaying or ineffective transforming structures and process as observed by SLF (DFID 1999).

As a result of the decay of these livelihood options, poverty levels have been witnessed as farmers dispose of their productive assets such as carts, ploughs and livestock to secure food from areas where it can be obtained. Climate change and variability tended to shrink both the natural and physical livelihood assets, further increasing the vulnerability of traditional farming systems. As a result, the farmers now depend on food aid programmes run by government and humanitarian agencies. Worsening poverty levels resulting from climate change, according to FGDs, have seen increasing migration from the three districts, particularly of the young active population. The dominant migration trends target South Africa and Botswana. Farmers alluded to increasing school dropout rates, as parents cannot afford fees anymore. Women farmers in the majority complained about early child pregnancies, especially in gold-resourced areas in the study sites, the outbreak of sexually transmitted diseases and tuberculosis, reports of increasing child trafficking incidences, prostitution and related negative coping mechanisms. These social ills are a sure indicator that traditional farming practices are no longer sustainable because of climate change, in terms of promoting increasing food security and viable livelihood options. In line with the SLF (DFID 1999), the three districts lack the human capital or assets, expressed in terms of knowledgeable farmers, that can see opportunities in their climate-distressed environment. The active labour force has shifted to neighbouring countries, leaving the elderly farmers further exposed to climate hazards. Farmers in the study districts acknowledged that they lacked the necessary education and skills to tackle climate threats. These human and social livelihood assets as propounded by the SLF (DFID 1999) require increased investment by the government and other development partners, if traditional farmers are to reduce their vulnerability to climate hazards.

#### Annual rainfall reduction resulting from shifting periods

According to the farmers in the three districts, their maize crops suffer moisture stress almost every season because of persistent drought. Maize is generally a water-loving crop, but given that maize is their staple crop, the farmers continue to plant it in spite of the changing climate patterns. The practice points to a lack of knowledge and awareness on the crop varieties that are likely to boost food security even in drought times. To counter the effects of climate change, some farmers are now into millet and sorghum production, and those who plant these small-grain crops indicated that they get a fair harvest from their practice. The need for transforming structures and processes as explained by the SLF (DFID 1999) seems to be slow and minimal, hence the low uptake of small-grain crops. Farmers indicated that both government and other development partners like NGOs were in the process of promoting small grains, but that the challenge was the lack of seed inputs for the small-grain crops, hence the continued cycle of food insecurity, lack of income for farmers and increasing vulnerability. Small-grain crops withstand drought, hence their appropriateness to the study sites. The farmers (34% across the three districts) explained that the low uptake of small grain crops was largely because of their difficulties in processing and the fact that the crops attract quelea birds, which prey on the crop before it is harvested. Crop losses, although largely because of climate change and variability, were also caused by poor storage, pests and diseases, prey animals and birds, as deduced from the focus group discussions. Further probing during the focus group discussions proved that some farmers lacked the know-how in terms of planting small grains, hence the low yields obtained. Scaring away birds from their fields was not only hectic but demanded extra labour that the majority of farmers did not have as a result of increasing migration owing to climate change. Small-grain crops such sa millet and sorghum have the potential to address food insecurity challenges amongst traditional farmers, given that they are drought resistant. Farmers will have to develop new strategies and technologies for scaring away birds, processing small grains and reducing yield losses resulting from poor storage, pests and diseases. Some 70 farmers out of 129 indicated that they lost on average 10 cattle per household per year because of decayed pastures, owing to reduced rainfall that impacted on pasture availability. CCare (2012) posited that the majority of farmers in Mangwe and Matobo had on average six head of cattle, having lost the bulk of their livestock (goats and cattle) to droughts in the 1990s and 2000s. Cattle are a physical livelihood asset that farmers rely on to complement their food requirements and generate income to meet other family needs such as fees for children, shelter, clothing and bride prizes (*lobola*), amongst many other uses (DFID 1999). Farmers in the three districts alluded to the fact that they can easily convert cattle to financial assets to make up for other family and community needs. The dwindling livestock herds, according to some farmers in the study sites, meant increased food shortages, as cattle would be sold to source food from wherever it could be found. Therefore, livestock deaths further exposed traditional farmers to climate threats and poverty.

One farmer in Matobo District remarked:

‘It is now scary to plant early, because one is not sure of the start of rainy season. The crop will be a write-off and chances of late receiving rains are minimal.’ (Farmer E, age 46, Makasa Ward)

The failure to do early planting, as is the tradition with subsistence farmers, compromised their food security across the districts. Most farmers felt that the changing weather patterns, particularly protracted droughts, meant gambling with one’s farming inputs like seed. Failure to do early crop planting or planting early would yield similar results – food shortages. About 45% of traditional farmers reacted to this phenomenon by abandoning crop production in favour of off-farm activities. Most smallholder farmers with very few or no technical farming skills reported the challenge of reduced acreage in terms of crop farming in the past 20 years, owing to reduced precipitation during the farming seasons over the years. Traditional farming practices like early crop planting are less viable without a permanent water source. One senior village head from Mangwe District quizzed:

‘Why should I continue ploughing my whole field, given unreliable and intermittent rainfall? One is never certain about climatic conditions anymore. I see no reason to continue tilling the whole field in the wake of shifting climatic patterns.’ (Village Head A, age 77, Mangwe District)

The farmer further explained that he did not appear in food aid registers because he had more than five cattle, and according to the criteria used for food aid, households with more than five cattle were considered well off and were not considered to benefit from food aid programmes. He went on to explain that traditional farmers with five or more cattle suffered food insecurity like everybody else, because they were affected by the same climatic conditions. He further added that their livestock fetched very low prices in the market, given their poor quality as a result of poor pastures. From the low prices fetched from the sale of their livestock, traditional farmers were not in a position to purchase supplementary feed for their livestock, given that the prices were beyond their reach. They preferred instead to use the little income from their animals to buy the little food they could find in the market, meaning that their few animals were left to starve in anticipation of a better coming season. They were not therefore well off compared with those who had no cattle. Some farmers noted that food aid programmes started very late in April, and by then households would already be starving, unless they got remittances from their relatives in South Africa and Botswana. There is need for education and training in livestock management in the wake of climate change. Investment in small livestock such as rabbits, goats and sheep is necessary, for these can easily adapt to drought-prone environments. Farmers ought to change their attitudes towards the small livestock and embrace them as a climate-adaptive measure, to ensure sustained food security in the three districts studied. The encroachment of acacia woodlands was observed across the three districts, and these could provide an excellent opportunity for investment in small livestock, as this type of vegetation is palatable to goats and sheep. Transforming structures and process (as expounded in the SLF), both on the institutional level (central government) and at the community and household levels, should be prioritised to reverse the vulnerability trends amongst traditional farmers in Matabeleland South Province.

The village head echoed the feelings of many a farmer in Mangwe District, a few (about 11%) of whom have abandoned field cropping in favour of nutrition gardens, raising small livestock such as rabbits and chickens. Increased mulching in nutrition gardens reduces evaporation rates and ensures soil moisture for vegetables for elongated periods. Small livestock such as goats and rabbits are well adapted to drought conditions as they use very little water and eat whatever little vegetation is available for them. Chickens and rabbits also feed from vegetable waste from nutrition gardens, thus effectively utilising the little food available and mitigating against climate change and variability. From the sale of nutrition garden proceeds (variety of vegetables) and small livestock, famers would get income and buy maize meal to ensure food availability for their households. The researchers also observed that most farmers continued to plant water-loving maize varieties, despite the water scarcity. The farmers indicated they experienced perennial crop losses because of moisture stress; a gap in climate knowledge was evident. Maize, according to farmers across the three districts, is the most preferred crop, as it is their staple food and is easy to process in terms of value addition. Moreover, most study participants spoke of the ‘better taste’ of maize meal, compared with meal from other grains. Continued planting of maize (staple crop) would require increasing investment in physical assets like dams for irrigation purposes and heavy financial injection into the sector to procure irrigation equipment such as sprinklers, solar panels and related production inputs. An increase was observed in the number of nutritional gardens along major rivers, like the Shashani in Matobo, the Semukwe in Mangwe and the Mtshabezi in Gwanda. Some farmers were now opting for irrigated nutritional gardens, as opposed to over-reliance on natural rainfall. Observed across the three districts was that nutritional garden residue was used to feed indigenous chickens and rabbits for the few farmers who had adopted these alternative farming practices. Irrigation schemes would mitigate climate challenges as experienced by traditional farmers and would improve food security in the three districts. A variety of vegetables that included leafy vegetables, tomatoes, onion, butter nuts and many more would be processed to prolong their shelf-life, hence increased income for traditional farmers in the light of climate threats.

The rainy season has gradually shifted, according to farmers during the focus group discussions. The shifting rainy season (from around January to May, as opposed to the known season from October to April) complicated the cropping calendars and compromised the farming season plans for both cropping and livestock breeding. This is a drastic shift from the known season where November was a wet and a cropping month, characterised by excellent pastures for both smaller and bigger livestock. The rainy season in Matobo is usually intercepted by four to five dry spells, causing many crop and animal losses (Farmer D, age 65, Sigangatsha Ward, 2017). The shifting rainy season results in uncertainties and confusion as to when to plant, what to plant and when to facilitate the mating of their livestock. It would appear from the discussions with farmers that the intermittent dry spells during the rainy season are now an almost permanent feature. Such dry spells come at a critical period of plant growth and calving/kidding and cause animal and crop losses as a result of reduced pastures and soil moisture, as indicated by the majority of farmers. The shifting seasons coupled with low farmer capacity and knowledge have compromised the farming output, particularly with regard to cropping and bigger livestock. Such developments further build up evidence of changing climate in Zimbabwe. Lack of appropriate climate change knowledge and related adaptation and mitigation strategies as signified by the low human capital in the SLF (DFID 1999) pose challenges in attempts to promote enhanced climate adaptation strategies in the districts studied.

#### Worsening droughts

El Niño events have become more frequent, persistent and intense in the last 20–30 years compared with the previous 100 years (Finlayson et al. 2006). Hence, El Niño conditions have increased the severity of droughts in southern Africa. In Zimbabwe, the five driest years on record have occurred since 1987. Since 1990, the country has experienced more than nine of the worst droughts in history, and the current trend is that droughts occur on a 3–5-year cycle (Zambuko 2010). This observation in the literature concurred with what farmers in the study sites cited as the effects of climate change and variability. Traditional farmers alluded to the fact that the farming seasons were no longer predictable and planting dates were missed, resulting in prolonged acute food insecurity, diminishing pastures, the death of livestock, environmental degradation and lowering of the water table.

Such physical manifestations of protracted droughts have effects even on the social fabric of communities. Social ills as pointed out by farmers during the focus group discussions included water and pasture conflicts, land invasions, teenage pregnancies, school dropouts and outward migration. Study participants alluded to the fact that droughts have driven the young men and women out of their communities in search of better prospects outside Zimbabwe. Tshuma and Mathuthu (2014) and Ndlovu (2016) cited similar social ills as emanating directly or indirectly from worsening drought events for the Mangwe and Matobo communities respectively. Continued outward migration, particularly of the young, active population, robs the studied districts of their future potential (human and social assets) (DFID 1999). The researchers observed that the majority of farmers in the three districts were the elderly, who were too weak to work and attain or absorb new climate-smart strategies for both crop and livestock production. Droughts disrupt traditional farming livelihoods that rely on rain-fed agricultural practices, given the lack of reliable water sources to facilitate food production under irrigation. Traditional farmers claimed that they were left with very few opportunities for earning a living, hence the outward migration triggered by climate change and variability. Investments locally in alternative livelihood options or interventions like training in small business initiatives would ensure that the young people can explore and exploit opportunities that are brought about by climate-related threats such as protracted droughts. Opportunities would include solar-driven energy for both irrigation and home use, biogas (energy generation), drip irrigation and conservation farming. Outward migration deprives the local communities of the much-needed labour to till the land and manage the livestock.

The researchers observed that farmers in Matobo District drove their livestock, especially cattle, onto private farms and Matobo National Park to mitigate against pasture challenges as a result of protracted and recurrent droughts. The farmers and the Matobo agricultural extension officer concurred that such cattle movements triggered increasing conflicts between the traditional farmers, the commercial farmers and the national park wardens. Extension workers at the three study sites also spoke of the prevalence of human–wildlife conflict as an increasing concern brought about by drought-related hazards, a factor of climate change and variability. Farmers in Matobo, for example, argued that given the negative impacts of climate change, the government could not prioritise wildlife over people, hence their justification for invading the National Park. The farmers further claimed that they felt they had a stake in the vastly protected resources such as National Parks, conservancies and forests. Zambuko (2007) seems to support this argument by farmers when he claims that soils in Matabeleland South Province are not only poor but have a low water-holding capacity, facilitating increased flash floods, leaving very little moisture for crops and rangeland. Hence, communities tend to violate the standing regulations that protect wildlife and forests and to invade protected areas to sustain their livelihoods.

#### Destruction of farming infrastructure

After Cyclone Eline in 2000 came Cyclone Dineo in 2017, with devastating impacts on the infrastructure that supports traditional farming activities. Infrastructure such as dams, roads, bridges, dip-tanks, schools, electric pylons, roads and boreholes were damaged, thus impacting negatively on local livelihoods such as nutrition gardening, transportation of agricultural inputs and buying and selling of both farm and non-farm goods. These physical assets, which are meant to support traditional farming, were destroyed by tropical cyclones, further exposing the communities to increasing food insecurity. Farmers across the three districts studied indicated that they could not access agricultural inputs in good time as the roads were destroyed, and they were cut off from transportation networks for a long time. As a result, it was also difficult to consult the extension officers to get advice on both crop and livestock pests and diseases. The agricultural extension officer in Matobo District had this to say about the cyclone impacts:

‘For over a month, farmers could not work their fields due to water-logging. Roads became impassable and bridges destroyed, making it difficult for farmers to access services.’ (Extension Officer 1, male, 37 years)

During the focus group discussions, the farmers indicated that dam walls were washed away; thus, livestock later lacked access to drinking water, resulting in farmers’ increased losses of animals. Commercial irrigation schemes also lacked water because of dam wall destruction resulting from the cyclone. The destruction of dip-tanks complicated livestock tick-borne disease surveillance, and many cattle succumbed to water-related diseases. Power for the running of engines in irrigation schemes was curtailed, as electricity pylons were uprooted. Famers could not communicate via mobile phones and land lines for elongated periods and could not solicit livestock medication and other agricultural inputs from relatives and extension services because of destroyed communication infrastructure. The importance of these physical assets in promoting traditional farming initiatives cannot be underestimated. The destruction of physical assets (DFD 1999) reversed all the gains the traditional farmers would have achieved, causing anxiety amongst many a farmer. Some farmers expressed that it was this destruction of infrastructure that caused them to scale down their farming activities, citing the uncertainties related to unpredictable weather patterns. The effects of Cyclone Dineo are still being felt to date, as very little has been done to ‘build back better’ (Sendai Framework 2015–2030). The researchers observed that across the three study sites, roads, dip tanks and dam walls remained unrepaired. The absence of these community physical assets is a detriment to mitigation of climate change and variability in the study sites. Improved engineering, technology and heavy investment in the rebuilding of critical infrastructure will help revitalise traditional farming systems.

### Climate information

One observation made by the researchers during the discussions with farmers was that keeping records of the destroyed infrastructure, amounts of rainfall and number of cyclone days was a challenge. It was difficult to discover the number of bridges and dams destroyed by the cyclones, as the farmers could not easily remember. Further probing established that the farmers did not keep records of the cyclone days and the amount of rainfall received in their respective areas. The implication here was that for traditional farmers, such information did not feed into their farming plans, a weakness amongst many a traditional farmer according to researchers. This article posits that records provide for invaluable climate information over time, which the current farmers do not have. A village head in Mangwe had this to say concerning record-keeping:

‘I do not keep records about cyclones and climate changes, and I think I speak for the majority of farmers.’ (Village Head, male, 67 years)

The village head went on to elaborate that their community relied on the natural environment (the behaviour of birds, insects, trees, wildlife, livestock, the stars and the moon) to interpret current and future weather patterns. Given this scenario, farmers in the communities saw no reason to keep records, as nature does that for them. It was observed that although this was true for the older farmers, the generally few younger farmers did not believe in the traditional climate early warning systems. The behaviour of the natural environment was not further probed, as the researchers felt this was a task for another day. About 75% of the farmers in Mangwe acknowledged during the focus group discussions that they did not keep records of the cyclone days, amounts of rainfall and related climate information. They indicated that they did not think it important as they hardly used records for their planning purposes. This article is of the view that without proper climate information, agricultural planning would not be effective in the light of climate change. This would weaken farmers’ adaptive and absorptive capacities and prohibit transformative practices in the three districts. Although a few farmers claimed that they relied on schoolchildren for climate information, an observation made by the researchers was that whilst many schools did not have weather stations, the few that had weather stations showcased outdated equipment that was in a state of disuse. There were no strategic meteorological centres within the communities. Although each of the districts has an official weather station, these were located at the central administration and business centres, far away from smallholder farmers and serving mostly the commercial farmers who can afford weather and climate information at a cost. The traditional farming systems scarcely benefit from the district weather stations. According to the farmers, this was a historical imbalance; the weather stations only served the interests of the colonial farmers. The discussants complained of a lack of appropriate services, including from the Meteorological Services Department. Farmer F (aged 58) in Gwanda was of the view that weather stations were alien to the locals, as they benefitted mostly commercial farmers. She added:

‘Even the weather bulletin as communicated over radio does not make much sense to us as it is not specific to our area. The weather information cannot be depended upon for planning purposes.’ (Farmer F, 58 years, Gwanda, 2017)

Over 60% of the farmers indicated that they had no radios and therefore depended on their traditional climate early warning forecasts for agricultural planning purposes. The impression given across the three districts was that traditional or indigenous climate early warning knowledge was more dependable than modern forecasts. Intensified education and training around the area of climate early warning would provide for proactive planning on the part of traditional farmers in the light of climate change and variability. Traditional farmers should be trained in taking, keeping and sharing weather information with the rest of the farming communities on platforms appropriate for all farmers.

### Climate change and environmental issues

Farmers cited that the effects of climate change and variability accelerated soil erosion caused by increasing flash flooding. The recurrent droughts explained the disappearance of pasture for livestock observed across the three districts. In Matobo and Gwanda districts, farmers had witnessed the invasion of certain tree species, including lantana camara, that displaced local grasses and bushes. The researchers observed hectares of bare land, devoid of vegetation and pasture. Bad land creation (gullies), although not a direct result of climate change, was facilitated by increasing gold panning and deforestation as farmers sought alternative livelihood options (gold panning and cutting trees for firewood that was sold in nearby towns and growth points). Such activities were embarked upon by farmers as direct responses to climate-devastated livelihoods. Farmers spoke of a general lowering of the water table; thus, water for domestic use, watering livestock and irrigation became a challenge. Accelerated soil erosion from flash floods compounded water challenges through siltation of dams and rivers.

Farmers cited communal management of forests and pasture land as challenges beyond individual efforts, because these were communally owned. Community social structures had decayed over the past 20 years and no one seemed to bother about communal approaches to natural resource management in the wake of climate change and variability. From the perspective of the researchers after carefully analysing the responses from the farmers, though, it was evident that a general lack of knowledge about climate change was the major reason behind poor management of natural and man-made (physical) resources. For example, instead of improved management of the few water resources available, farmers drove their livestock over long distances for watering, thus causing the livestock a lot of stress and attrition in the process. It also meant that households had less water to venture into nutrition gardens to supplement their failed field cropping. The size of livestock herds has been drastically reduced, with 61 farmers out of 129 (47.3%) mentioning having abandoned cattle rearing because of the prolonged lack of pasture and environmental degradation in general.

Chief Malaba (76 years) and Chief Bango (77 years) of Matobo and Mangwe districts, respectively, also complained of increasing vulnerabilities caused by environmental degradation (soil erosion, siltation and disappearance of pasture), stating that these impacted negatively on annual crop and livestock production. Livestock has been the mainstay of the people’s livelihoods in Matabeleland South Province communities for centuries, and livestock decimation means that people are now highly exposed and their resilience eroded. Chief Malaba added:

‘I had to request for an additional farm allocation 50 km away from Sigangatsha Ward, so that people could feed their livestock, given the disappearance of pasture and general environmental degradation. I even have applied to government for a restocking programme in the district as over 75% of farmers had lost their cattle to drought.’ (Chief Malaba, 76 years, Matobo District)

More needs to be done in addition to more land allocation and restocking programmes. The need for education and training, especially in terms of climate change knowledge and general natural resource management, should not be left to extension officers only but should be extended to poor, vulnerable farmers. Losing this physical livelihood asset (cattle) exposed farmers and their households to future climate hazards. These find support from observations by Practical Action (n.d.); Zambuko (2007). Crop failure and livestock deaths have been a result of water scarcity during long drought periods or water logging during tropical cyclones and flash flooding (Brown et al. 2012). Grazing lands have been decimated through soil erosion and invasion of alien vegetation species like *Lantana camara*, with very few prospects for veld restoration, according to farmers in Mangwe and Matobo districts. Alternative off-farm activities like brick moulding, a direct response to diminishing water resources for farming caused by climate change, has promoted increasing deforestation as people cut down trees to season their bricks. This practice, according to farmers especially in Gwanda, has triggered conflicts amongst villagers, especially between those who still have large herds of livestock and those who have lost livestock. These conflicts, according to farmers in these districts, have their roots in climate change impacts, in that recurrent droughts, for example, continue to reduce the amount of pasture land available in the study districts. Ill-advised livelihood activities in the study sites further expose farmers to climate hazards, hence the need to develop livelihood assets as advised by the SLF (DFID 1999).

The research team also observed small herds of cattle, of about 12 on average, dotted across the districts because of climate change losses. Larger herds, according to the farmers, had been driven to areas along the border with Botswana or towards Bulawayo for temporary grazing during lean pasture periods. These are protected areas; some are conservancies, whereas some are commercial ranching areas abandoned after the land reform exercise in 2000. Such semi-pastoralism was common amongst those considered ‘well off’ by community members, for they could hire herders to take care of their large herds. The practice was further explained by extension officers as a climate adaptation strategy to save livestock, but a poor strategy in that farmers tended to lose their livestock to diseases during transit. Semi-pastoralism is not a sustainable strategy as a climate adaptation measure. The practice further degrades virgin lands that could be used effectively as part of the ecosystem services in mitigating climate-related challenges. Although this pastoralism was part of the traditional farming system in the 1970s, it was discontinued because of increasing population on the land. Reviving the strategy or intervention has sparked a great deal of conflict between semi-pastoralists and landowners, implying that the strategy is not suitable under the current conditions.

Sprouting vegetation and grasses and coppiced trees are at their weakest point at this period; thus, the vegetation cover and foliage for livestock and wildlife are badly affected. Communities remain food insecure; their livestock lack grazing at this critical period when they are recovering from the effects of the dry season, an indication of weak resilience structures and less viable farming practices. Some farmers indicated that they procure stock feed after disposing of a few animals, instead of using the money to send their children to school. Farmers with access to big rivers, such as the Semukwe in Mangwe, the Shashane in Matobo and the Tuli in Gwanda, reported that they dug deep wells in the river beds to provide water for both household usage and to water their livestock, an exercise that is labour intensive. Extension officers in the three districts were concerned about the disposal of productive household assets such as cattle, scotch carts, ox-drawn ploughs and hoes to secure food and stock feed by many rural farmers. Disposal of such critical assets further exposed the traditional farming systems, which mainly depended on cattle for draught power; for example, with limited draught power, the capacity of the vulnerable farmers to fully utilise the little rainfall received was eroded. Agricultural productivity for traditional farmers in Mangwe, Matobo and Gwanda districts has been greatly weakened as a result of the unpredictable mid-season dry spells.

According to farmers in Mangwe and Matobo especially, the disappearance of certain grass and tree species was worrisome. This development as explained by famers forced their animals to travel across a vast terrain in search of grazing, exposing their livestock to contracting diseases such as anthrax and black leg along the way; IPCC (2007b) supports this finding when alluding to the fact that various vegetation types and grasses, critical for livestock feed and in comprehending climate trends and predictions, disappear because of climate change forces. With increasing water scarcity because of environmental degradation, and the disappearance of pastures, come numerous human conflicts in the studied districts (Tshuma & Mathuthu 2014; WFP 2006). According to the extension officers, large-scale food aid and development programmes, such as livestock feed distribution and tree-planting operations by both government and NGOs, are likely to continue. Communities in Mangwe, Matobo and Gwanda have sunk into perennial receivers of food and developmental aid because of the diminishing livelihood options. Farmers in Matobo indicated that they barely harvest enough to feed their families, hence their reliance on food aid. In Gwanda and Mangwe, farmers indicated that they lost between 10 and 15 cattle per household as a result of diminishing pastures owing to climate change and variability. The reduction in physical assets, both at the individual household and community levels, impacts negatively on the economy at both scales, undermining the resilience of traditional farmers to climate change and variability. The well-being of farmers as proposed by the SLF remains weakened as long as asset development is not prioritised.

Increasing environmental degradation resulting from climate change and variability has rendered over 75% of the rural population exposed to epidemiological and human-induced hazards in Matabeleland South Province (Ndlovu 2011; Oxfam Canada 2013). The findings are further supported by the United Nations Development Programme (UNDP) and United Nations International Strategy for Disaster Reduction, when they point to the devastating droughts of 1982, 1992, 2002, 2006 and 2011 that left a trail of crippled livelihoods across Matabeleland South Province and Zimbabwe in general (UNDP 2009; UNISDR 2004). It was deduced from the focus group discussions that Matabeleland South famers were failing to spring back to productivity because of a lack of capacity to deal with climate change threats (droughts, mid-season dry spells, flash floods, heat waves and frost). Farmers acknowledged that their major livelihood activities – rain-fed crop farming and livestock rearing – were near decimation. Favourite crops like maize continue to be a write-off every season because of the siltation of water bodies such as dams and rivers. Crop diversification and the introduction of small livestock are interventions that farmers could adopt to mitigate climate-related hazards.

Invasion of species like *Lantana camara*, species relocation and desertification were observed by the research team across the districts. Ecosystems are critical in contributing to biodiversity and human development (Biggs et al. 2004).The species tended to favour the new climatic conditions over the study areas. Farmers attributed these challenges to failure by plants to adapt to climate changes (FGDs 2017). In support of this finding, project that semi-arid areas in Africa will increase between 5% and 8% by 2080. Land degradation, poverty, population pressure and lack of structural transformative interventions as expounded in the SLF are inherent within traditional farming systems and will continue to expose farmers to climate change disasters, even as observed by the Food and Agriculture Organization (FAO; 2007) and the United Nations Environment Programme (UNEP; 2011).

It was deduced from the discussions that farming households in the study districts were labour constrained, making it difficult to break the cycle of poverty. There are limited adaptive capacities amongst the poor, the sick, the elderly and woman- and child-headed households, as also observed by Musarurwa and Lunga (2012) and UNDP (2009). In support of this notion, the Zimbabwe Vulnerability Assessment Committee (ZimVAC; 2014) observed that 51% of households in Matabeleland South Province had 49% of labour available for normal agricultural activities against a national average of 63%. (Farmers indicated that labour challenges make it difficult for subsistence farmers to reach their potential without outside assistance.)

### Climate adaptive measures in the study districts

In trying to mitigate climate change impacts in the study sites, the government, through its drought policy, discouraged food aid interventions that created dependence. The new interventions integrated the development of community assets through food-for-work or cash-for-work initiatives (Government of Zimbabwe 2007; Ncu 2005). Assets developed in Mangwe, as stated by the communities, included the fencing of small dams, construction of earth dams, schools and road repairs, paddocking, reforestation and gully reclamation, a programme funded and implemented by Practical Action in 2016 (Practical Action n.d.). It was envisaged that the restoration of such community assets would motivate alternative livelihoods for the affected communities. The logic behind it is to promote community-managed climate interventions through enhancement of community assets (Christian Care, 2012; USAID, 2017). Non-governmental organisations operating in the three districts complemented government efforts by providing the resources to kick-start and sustain such interventions.

The farmers indicated that NGOs such as Dabane Trust and Christian Care focussed on borehole drilling, small dam construction and the establishment of nutritional gardens, although World Vision and Khulasizwe Trust facilitated disaster risk-reduction education, conservation farming and small livestock promotion as adaptation measures. According to the extension officers, the awareness drive and investment in water source development by NGOs were meant to promote smart adaptive agricultural initiatives to improve food security and get the communities to diversify their livelihoods in the study districts. Such interventions are necessary to provide for transformative strategies and processes (SLF) to get the traditional farmers out of their vulnerable situation. It is envisaged that such strategies will usher in improved agricultural productivity, improved well-being of the farmers and their households and facilitate increased incomes that will then provide the much-needed insurance against climate disasters. With cash from improved agricultural productivity and livelihood diversification, traditional farmers would source food and small livestock from wherever these could be found to mitigate climate threats. Earth dams and deep wells facilitated by Dabane Trust and Christian Care did bring temporary relief as communities ventured into irrigated nutritional gardens, rabbit keeping and poultry, realising income thereafter, according to the farmers during discussions. Key informants from NGO staff added that disaster risk-reduction education through farmer-field schools, look-and-learn visits and field days helped increase the uptake of climate-smart practices such as conservation farming, rainwater harvesting techniques and increased use of solar energy. The key informants further explained that such initiatives have, to a lesser extent, triggered some off-farm livelihoods such as voluntary savings and lending schemes for women, poultry, fishing, beekeeping and buying and selling across the three districts, even as observed by USAID (2014). Although these interventions were welcomed by the communities, the farmers during discussions were of the view that NGO interventions only targeted a few people, implying that the impacts of these interventions were not felt or appreciated by the larger communities. Increased targeting therefore would support the SLF in that investment in developing physical livelihood assets would help address community vulnerabilities to climate change and variability.

In Mangwe and Matobo districts, 30% of the farmers have adopted conservation farming techniques to counter climate change and improve food security. There is no tillage in conservation farming; farmers stated that they simply dig holes, add manure and plant seed when the ground has adequate moisture. Holes are meant to hold water, and this water is then made available to the growing plants. The technique also involves mulching the planted area to reduce moisture loss through evaporation, thereby conserving water in the soil to ensure that plants have adequate water for elongated periods. The initial adoption rates by the targeted traditional farmers, according to extension workers in the two districts, were low, given that conservation farming was introduced initially for the very vulnerable households who owned no draught power, a criteria adopted by NGOs. Non-governmental organisations envisaged that the initiative would promote the creation of new productive assets, such as the purchase of wheelbarrows, hoes and other related farming tools, including carts and livestock, for the most vulnerable households. In Matobo District, according to farmers, conservation farming focussed on the growing of drought-tolerant crops such as sorghum and millet, to drive communities out of food insecurity. Crop diversification translates into widening physical livelihood assets, thus giving farmers a wide choice as to what crop to produce. With increased mulching, farmers in Matobo indicated they realised 3–4 tonnes/hectare from their plots, compared with 0.4 tonnes/hectare before the adoption of conservation agriculture. One farmer from Matobo District concurred when he said:

‘Adoption of millet and sorghum ensured food security for my household in the past season. The two crops withstand drought conditions, and I would increase the acreage of these crops in the coming season.’ (Farmer G, age 67, Matobo District)

This finding was supported by CCare (2012), which cited increased yield per hectare for small-grain crops under conservation farming in Mangwe and Matobo districts. Factored into conservation farming are other climate adaptation innovations such as early planting, now that moisture is conserved in the soil through mulching, adoption of heat-tolerant crops (groundnuts, rapoko) and livestock (rabbits and indigenous chickens), improved pest management practices and analysis and dissemination of weather information for improved farming decision-making. The FAO (2001), in support of this finding, observed that the dissemination of weather-related information in local languages was critical in facilitating farming decision-making processes. Some farmers in Mangwe and Matobo districts indicated that they dug in-field water harvesting trenches or dead-level contours in their plots, climate-smart techniques that enabled them to collect and store whatever amount of rain they received in their plots. The collected water will slowly percolate into the soil on either side of the contour and provide the necessary moisture for crops over an elongated period. Nyathi (2008), in support of this finding, posits that dead-level contours provide the extended soil water availability period needed for crops to mature. The uptake of conservation farming, whilst pleasing in terms of the quality and quantity of the produce, has been very slow and at times abandoned prematurely for lack of faith and commitment to hard work.

Some farmers in Gwanda indicated that they had ventured into cattle fattening as a business, to enable them to maximise on their herds and reduce the impacts of climate change on their livestock livelihoods. According to the agricultural extension officer in Gwanda, this was meant to reduce overgrazing and improve cattle management practices for traditional farmers. Farmers reported that they had embraced livestock supplementary feeding, largely pan feeding and fattening supplements as an alternative climate-adaptation strategy. Improvements in livestock management through the adoption of climate-smart interventions like cattle fattening added to the dimension of financial, social and human capital or assets, all to protect farmers against the ravages of climate hazards. Farmers acknowledged that they had very little information on climate change and did not fully appreciate the effects and impacts on their cropping and livestock activities. They expressed a desire for regular information flow and registered the need for the establishment of a meteorological office in their area. During the FGDs, farmers felt that extension workers gave inaccurate weather information. In addition, the farmers indicated that whatever climate or weather information they received over the radio was never specific to their operational areas. The farmers suggested that they should receive training from the government and NGOs on using weather-forecasting gadgets, so that they would be in a position to read and interpret information for their counterparts. Older farmers insisted on the importance of traditional weather-forecasting practices and volunteered to teach young farmers and extension officers how the indigenous climate early warning system works. The farmers, as it seemed during the discussions, realised that they needed to adopt more appropriate climate-smart technologies such as conservation farming, water-harvesting from their rooftops and focussing on drought-tolerant crops and change their centuries-old practices, which are no match for current climatic extremes.

Farmers in Gwanda called upon government and non-state actors like NGOs and the private sector to support environmental and disaster risk-reduction clubs in schools. They hoped that schoolchildren would disseminate information to their parents and help to build climate-resilient communities. The idea is to ‘catch them young’, according to farmers, to facilitate and motivate attitude change and reduce future climate vulnerabilities. The farmers believed that if their children were taught about climate change in school, they would come home and share that knowledge with their parents, and according to them, this was likely to yield positive perceptions around the discourse on climate change.

## Conclusion

Traditional farmer perceptions of climate change in Mangwe, Matobo and Gwanda districts were varied. The farmers did well to associate delayed rainfall periods, increasing flood occurrences, mid-season dry spells, frost, increasing summer temperatures, warm winters and the outbreak of crop and livestock diseases such as anthrax, black leg and tick-borne illnesses with climate change and variability. Some misconstrued environmental degradation to climate change and variability, although the majority thought that the human factor in climate change was minimal. In other words, the farmers disassociated themselves from the causes of climate change. This is explained by low literacy and climate awareness levels on the part of traditional farmers. Musarurwa and Lunga (2012) seem to support this conclusion when they refer to low climate knowledge levels on the part of smallholder (subsistence) farmers in Mangwe and Lupane districts in Zimbabwe.

Farmers’ perceptions of climate change and its impacts on their livelihood options were not misplaced; they understood the linkages. The farmers experienced annual heat waves, protracted droughts, delayed rainy seasons, incessant tropical cyclones and flash floods and described these as impacting negatively on their natural, physical, human, social and financial assets as indicated in the SLF (DFID 1999). The decay of these assets explains the chronic food insecurity and poverty status of the traditional farming communities in Mangwe, Matobo and Gwanda districts in Zimbabwe. The implication is that, at current vulnerability levels, given the persistent food insecurity and acute poverty, the current nature of livelihood assets or capital is not sustainable. Frankenberger et al. (2000), in trying to promote the rejuvenation of these livelihood assets, posit that there is a need to strengthen the vulnerable communities’ adaptive, absorptive and transformative capacities to help build climate resilience in communities. For farmers in the study sites, it was evident that these three-tier capacities were weak or almost non-existent, hence the continued cycle of poverty and chronic food insecurity.

For the majority of farmers, climate change was associated with a great deal of environmental degradation, the siltation of dams, the disappearance of certain tree species and palatable grasses for their livestock, overgrazing, low and unreliable rainfall, delayed rainy season and mid-season droughts or dry spells. Crop and animal management gaps were also noted across the three districts, as farmers were still stuck with centuries-old practices such as keeping large herds of cattle, over-reliance on a single crop for their staple food and cropping only after receiving rainfall, in spite of the changes in climate. The absence of appropriate and relevant climate early warning systems further contributed to the vulnerability to mid-season dry spells and outbreak of both crop and livestock pests and diseases. This anomaly indirectly contributed to increased food insecurity and environmental degradation. The farmers hardly accessed official weather and climate information, and this compromised their farming calendars, hence increasing food insecurity in the three districts. In other words, farmers’ practices were not sustainable in the wake of unpredictable climate trends. Traditional farming systems are on the verge of collapse because of climate-related threats. Given that the smallholder farming system sustains the bulk of the population in Matabeleland South Province in Zimbabwe, accelerated education and training are needed to change farmers’ perceptions around climate change and its impacts on farming practices.

## Recommendations

Traditional farming systems such as mixed farming and extensive crop and livestock rearing remain the main livelihood option for most farmers in Mangwe, Matobo and Gwanda districts. Firstly, there is need for concerted efforts by the school system, relevant government institutions, the private sector and NGOs to embark on climate awareness education to enable local farmers to make informed decisions concerning their cropping and livestock production systems. Such education should integrate improved crop and livestock management curricula to foster attitude change and perceptions around the viability of both crops and livestock in the changing climate. Local farmer representatives should be seconded for a week or two to tertiary institutions that offer climate education, so they can engage in meaningful conversations with academics and professionals; information gathered during this period of engagement will then be passed to other farmers back home.

Secondly, the government should mainstream or integrate ‘climate change’ into the school curriculum from early childhood development to tertiary level. Teacher training institutions should as a matter of urgency prioritise majoring in climate change education, as well. This is to ensure future generations are capacitated to tackle climate change challenges from an advantage point. ‘Catching them young’ (children) at school will ensure that appropriate climate messages that would trigger action are sent home from schools. The Bindura University of Science Education and the National University of Science and Technology already offer Disaster Management and Development Studies. These institutions of higher learning could be springboards for curriculum development and research. Research institutions are obliged to work with traditional farming communities to develop further evidence of climate change and recommend viable and sustainable adaptive, absorptive and transformative climate-smart interventions to help boost agricultural productivity amongst traditional farming communities.

Thirdly, diversification of livelihoods would assist in mitigating climate change impacts. To realise this noble objective, traditional farmers (after intensified awareness education and training) should be encouraged by both government and development partners to turn their small plots and low livestock numbers into business units, supplying an established or organised market. In addition to cropping and livestock, they should be assisted to diversify into value addition of their produce and also consider off-farm activities as part of insurance against failure of certain livelihoods in the face of climate change and variability, spreading the climate change risk. Such development would ensure improved income for households (financial asset). Improved income would also promote investment in improved seed and livestock varieties tolerant to droughts, as well as investment in climate-smart technologies.

Fourthly, the private sector, through partnership with the government and traditional farmers should work at establishing community (village) digital weather stations (climate information centres). Local farmers, who would include some village heads, should be trained by the National University of Science and Technology in utilising digital weather stations, because the university is located closer to the farmers. From the Monitoring of the Environment for Security in Africa-Southern Africa Development Community (MESA-SADC) facility at the university, local farmers would then be able to share climate information specific to their villages with farmers back home, interpret the information and begin to use it for their agricultural planning purposes. Platforms for sharing climate information already exist within communities, and these include village meetings, schoolchildren and various agricultural fairs and farmer-field meetings. The university can adopt these communities and help them in their drive towards climate resilience. Village-based climate information centres, manned by trained local facilitators (farmers), will enhance timely and reliable information sharing for improved climate mitigation. The marrying of traditional climate knowledge to modern practices is highly recommended for enhancing climate resilience. The university is encouraged to do research on indigenous climate early warning systems together with local farmers and facilitate the mainstreaming of these into modern weather and climate forecasting systems.

These recommendations speak to policy changes in education, commerce and industry, climate and weather departments, and agriculture. Such changes would ensure that climate knowledge and education is afforded to current and future generations at the study sites. The approach is futuristic and promotes sustainability in the light of climate change and variability.
